# The Toll-Like Receptor 4 Antagonist Eritoran Protects Mice from Lethal Filovirus Challenge

**DOI:** 10.1128/mBio.00226-17

**Published:** 2017-04-25

**Authors:** Patrick Younan, Palaniappan Ramanathan, Jessica Graber, Fabian Gusovsky, Alexander Bukreyev

**Affiliations:** a Department of Pathology, The University of Texas Medical Branch, Galveston, Texas, USA; b Department of Microbiology and Immunology, The University of Texas Medical Branch, Galveston, Texas, USA; c Galveston National Laboratory, The University of Texas Medical Branch, Galveston, Texas, USA; d The University of Texas Medical Branch, Galveston, Texas, USA; e Eisai Inc., Andover, Massachusetts, USA; University of Maryland, College Park; University of Maryland, College Park

**Keywords:** Toll-like receptor 4, cytokine storm, Ebola virus, Marburg virus, viral hemorrhagic fever

## Abstract

The 2013-2016 outbreak of Ebola virus (EBOV) in West Africa, which has seen intermittent reemergence since it was officially declared over in February of 2016, has demonstrated the need for the rapid development of therapeutic intervention strategies. Indirect evidence has suggested that the EBOV infection shares several commonalities associated with the onset of bacterial sepsis, including the development of a “cytokine storm.” Eritoran, a Toll-like receptor 4 (TLR4) antagonist, was previously shown to result in protection of mice against lethal influenza virus infection. Here, we report that eritoran protects against the lethality caused by EBOV and the closely related Marburg virus (MARV) in mice. Daily administration of eritoran reduced clinical signs of the disease and, unexpectedly, resulted in reduced viral titers. Analysis of peripheral blood indicated that eritoran reduced granulocytosis despite an apparent increase in the percentage of activated neutrophils. Surprisingly, the increased survival rate and reduced viremia were not accompanied by increased CD3^+^ T lymphocytes, as lymphopenia was more pronounced in eritoran-treated mice. Overall, a global reduction in the levels of multiple cytokines, chemokines, and free radicals was detected in serum, suggesting that eritoran treatment may alleviate the severity of the “cytokine storm.” Last, we provide compelling preliminary evidence suggesting that eritoran treatment may alter the kinetics of cytokine responses. Hence, these studies are the first to demonstrate the role of TLR4 in the pathogenesis of EBOV disease and indicate that eritoran is a prime candidate for further evaluation as a clinically viable therapeutic intervention strategy for EBOV and MARV infections.

## IMPORTANCE

A hallmark of bacterial sepsis is the uncontrolled activation of the TLR4 pathway, which is the primary cause of the pathological features associated with this disease. Considering the importance of TLR4 signaling in bacterial sepsis and the remarkable pathological similarities associated with infections caused by filoviruses Ebola virus (EBOV) and Marburg virus (MARV), we assessed the ability of eritoran, a TLR4 antagonist, to protect mice against these viruses. Here, we show that eritoran effectively promotes survival of mice of filovirus infection, as 70% and 90% of mice receiving daily eritoran treatment survived lethal EBOV and MARV infections, respectively. Eritoran treatment resulted in a remarkable global reduction of inflammatory mediators, which is suggestive of the mechanism of action of this therapeutic treatment. These studies are the first to show the critical importance of the TLR4 pathway in the pathogenesis of filovirus infection and may provide a new avenue for therapeutic interventions.

## INTRODUCTION

Sporadic outbreaks of Ebola virus (EBOV) infections historically occurred in Central Africa (1), while the most recent and the largest epidemic occurred in 2013 to 2016 in West Africa, which resulted in over 27,000 human cases and over 11,000 fatalities (2, 3). The unprecedented scale of this epidemic has demonstrated the urgent need for therapeutic intervention strategies against Ebola virus disease (EVD). EVD has been shown to exhibit several hallmarks that are associated with bacterial sepsis or what is also known as “septic shock” (4–7). Multiple coagulopathies, including disseminated intravascular coagulation (DIC) due to the increased expression of tissue factor (TF), d-dimers, thrombomodulin, ferritin, and thrombocytopenia, are associated with both classical, bacterium-induced septic shock and EBOV-induced shock. Furthermore, immune dysfunction, lymphopenia, and systemic inflammation due to the onset of a mass, uncontrolled production of inflammatory mediators known as a “cytokine storm” are also observed during both EVD and bacterial sepsis (4, 5, 8, 9). Last, late stages of both EVD and bacterial sepsis are associated with endothelial dysfunction and organ failure (5, 7).

Development of sepsis caused by Gram-negative bacteria is associated with production of lipopolysaccharide (LPS), which activates the Toll-like receptor 4 (TLR4) pathway (10, 11). In addition, recognition of oxidized host phospholipids (Ox-PLs), which are produced following the accumulation of reactive oxygen species (ROS) upon exposure to LPS, has also been implicated as a secondary, yet potent elicitor of TLR4 signaling (12). Recently, Shirey et al. demonstrated that TLR4^−/−^ mice are resistant to lethal influenza virus infection (13). Furthermore, the TLR4 antagonist eritoran protected mice from lethal influenza virus infection. Activation of the TLR4 signaling pathway requires transfer of LPS molecules from aggregates to CD14, followed by transfer to the hydrophobic pocket of myeloid differentiation factor 2 (MD2) and engagement and dimerization of TLR4. Similarly to LPS, eritoran is a lipid A monomer that binds to MD2; unlike lipid A, eritoran does not induce TLR4 dimerization and activation, thereby antagonizing TLR4 (14). Although previous studies using cell lines have indicated putative interactions and the induction of the TLR4 signaling pathway by both EBOV glycoprotein (GP) and soluble GP (sGP) (15, 16), the implications of TLR4 signaling in the pathogenesis of EVD have thus far remained highly circumstantial. We therefore sought to determine the putative protective role that TLR4 antagonists may have on infections caused by the filoviruses EBOV and the closely related Marburg virus (MARV) in murine models. Here, we provide the first evidence demonstrating that the TLR4 antagonist eritoran is effective at promoting survival of mice exposed to filoviruses.

## RESULTS

**FIG 1:**
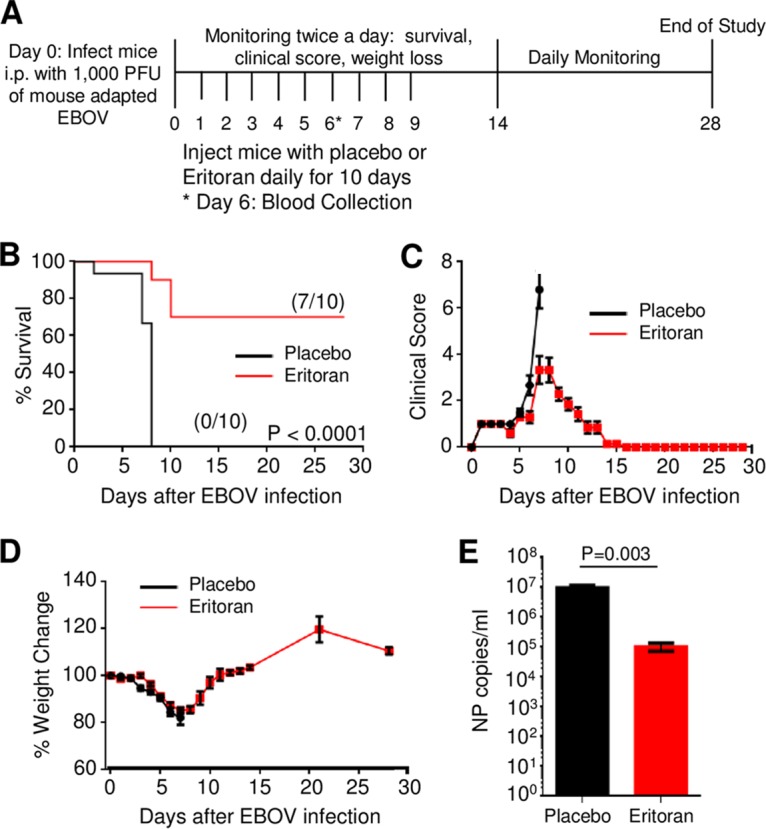
Eritoran protects mice from lethal EBOV challenge. (A) Overview of studies investigating the use of eritoran as a therapeutic for EBOV infection. C57BL/6J mice were challenged via i.p. route with 1,000 PFU of mouse-adapted EBOV. Mice received 10 daily injections of eritoran or placebo (vehicle) via the i.p. route. (B) Survival curves from two independent experiments consisting of groups of 5 mice per group. (C) Illness scores assigned as described in Materials and Methods. (D) Weight change following EBOV challenge. (E) Viremia on day 6 postinfection. (B to D) Mean values of two independent experiments of 5 mice per group ± standard errors. (E) Mean values ± standard errors based on 4 mice in placebo group and 5 mice in eritoran-treated group. See also Fig. S1 and S2 in the supplemental material.

On day 0, groups of 7- to 8-week-old C57BL/6J mice were infected with 1,000 PFU of mouse-adapted EBOV via intraperitoneal (i.p.) injection, which routinely results in 100% rates of death within about 7 days (17) (Fig. 1A). Starting immediately after infection (day 0), 10 consecutive daily 233-µg doses of eritoran were delivered by i.p. injections. Each mouse was weighed daily and scored for clinical signs of disease (see Materials and Methods). One hundred percent of mice receiving the placebo died or met criteria for euthanasia by day 8 after infection (Fig. 1B). Of the mice receiving daily eritoran treatment, 70% survived until the end of the study. The illness scores paralleled the survival curves, with mice receiving eritoran exhibiting reduced scores in comparison to placebo-treated mice beginning from the symptomatic stage of disease on day 6 (Fig. 1C). Weight loss was highly evident in both groups; however, eritoran-treated mice returned to normal weight following a period of recovery after day 8 (Fig. 1D). Quantitative reverse transcription-PCR (RT-PCR)-based analysis demonstrated that eritoran treatment reduced viremia by 2 logs (from 10^7^ to 10^5^ genome equivalents per ml) (Fig. 1E). Based on these findings, we surmised that disabling of TLR4 signaling would increase survival. However, TLR4^−/−^ mice remained as susceptible to EBOV as wild-type (wt) mice (see Fig. S1 in the supplemental material). Moreover, short-term treatment with eritoran (days 0 to 3) or delayed treatment starting at 48 h postinfection did not improve the rate of survival (Fig. S2).

10.1128/mBio.00226-17.1 FIG S1  EBOV infection of TLR4^−/−^ and TNFR1/2^−/−^ mice. Wild-type (wt) C57BL/6J, TLR4^−/−^, and TNFR1/2^−/−^ mice were challenged with mouse-adapted EBOV. (A) Survival curve. (B) Clinical scores. (C) Weight change, percent. Download FIG S1, PPT file, 0.1 MB. Copyright © 2017 Younan et al. 2017 Younan et al. This content is distributed under the terms of the Creative Commons Attribution 4.0 International license.

10.1128/mBio.00226-17.2 FIG S2  Testing of reduced duration and delayed eritoran treatment. C57BL/6J mice were treated with eritoran or placebo on days 0 to 3 (A to C) or 2 to 9 (D to F). (A and D) Survival curves. (B and E) Clinical scores. (C and F) Weight. Mean values ± standard errors based on 5 mice per group. Download FIG S2, PPT file, 0.2 MB. Copyright © 2017 Younan et al. 2017 Younan et al. This content is distributed under the terms of the Creative Commons Attribution 4.0 International license.

**FIG 2:**
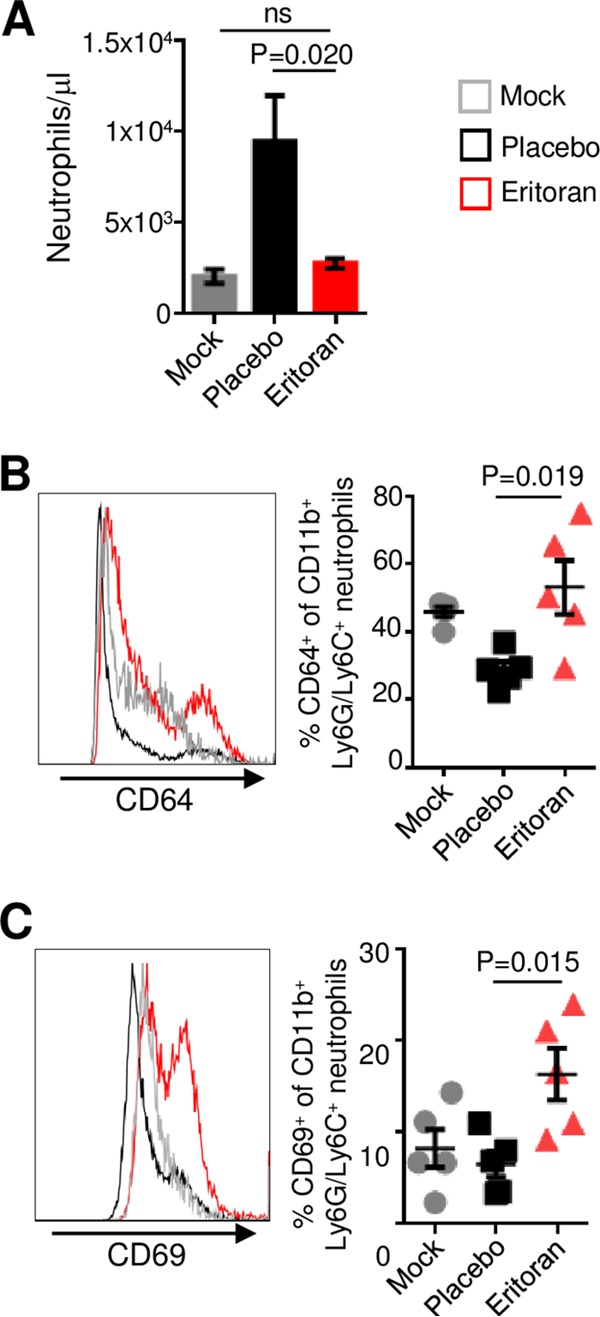
Eritoran treatment increases neutrophil activation. Analysis of peripheral blood in mock-infected, placebo-treated, and eritoran-treated infected mice by flow cytometry. (A) Absolute neutrophil counts. (B) Percentages of CD11b^+^ neutrophils positive for CD64. (C) Percentages of Ly6G/Lyc6C neutrophils positive for CD69. See also Fig. S4.

Flow cytometry-based analysis demonstrated an increase in the amounts of granulocytes from 2,070 cells/µl in uninfected mice to 9,485 cells/µl in infected placebo-treated mice, suggesting the development of granulocytosis (Fig. 2A). However, treatment with eritoran reversed this process, resulting in 2,786 cells/µl. Further analysis of the gated granulocyte subset (Fig. S3) revealed that the majority of this population in both groups was double-positive CD11b^+^ Ly6G/Ly6C^+^ neutrophils (Fig. S4). Intriguingly, treatment with eritoran significantly increased the percentages of neutrophils expressing the activation markers CD64 (Fig. 2B) and CD69 (Fig. 2C), despite the sharp reduction of the total number of granulocytes. Taken together, these findings indicate that treatment with eritoran reduces granulocytosis and results in a higher percentage of activated CD11b^+^ Ly6G/Ly6C^+^ neutrophils.

10.1128/mBio.00226-17.3 FIG S3  Changes in peripheral blood white blood cells. Analysis of white blood cells following red blood cell lysis of EBOV- and mock-infected eritoran- and placebo-treated mice by forward (FSC) and side (SSC) scatter demonstrating increased granulocytosis and alterations in the relative ratio of granulocytes, lymphocytes, and monocytes (MO). Flow plots are representative of one of 5 mice for mock-infected and eritoran-treated mice and one of 4 mice from the placebo group. Download FIG S3, PPT file, 0.1 MB. Copyright © 2017 Younan et al. 2017 Younan et al. This content is distributed under the terms of the Creative Commons Attribution 4.0 International license.

10.1128/mBio.00226-17.4 FIG S4  Percentages of CD11b^+^ cells positive for Ly6G/Ly6C^+^. Mean values ± standard errors based on 5 mice per group. NS, no significant difference. Download FIG S4, PPT file, 0.1 MB. Copyright © 2017 Younan et al. 2017 Younan et al. This content is distributed under the terms of the Creative Commons Attribution 4.0 International license.

**FIG 3:**
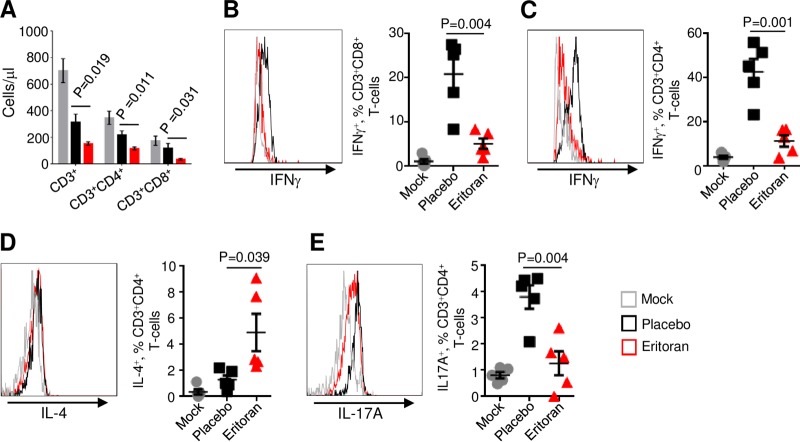
Eritoran reduces expression of Th1- and Th17-associated cytokines by T lymphocytes. (A) Absolute counts of CD3^+^ T-cell subsets in white blood cells. (B to E) Percentages of the indicated T-cell populations positive for the indicated markers of activation. (B) CD3^+^ CD8^+^ T cells positive for IFN-γ. (C) CD3^+^ CD4^+^ T cells positive for IFN-γ. (D) CD3^+^ CD4^+^ T cells positive for IL-4. (E) CD3^+^ CD4^+^ T cells positive for IL-17. Mean values ± standard errors based on 5 mice per group on day 6 postinfection. Histograms are representative of one mouse from each group. See also Fig. S5.

We next examined CD3^+^, CD3^+^ CD4^+^, and CD3^+^ CD8^+^ T-lymphocyte subsets. Infection with EBOV resulted in the reduction of all these populations, which is consistent with lymphopenia, a hallmark of EBOV infections in both humans and animal models of EVD (reviewed in reference 18) (Fig. 3A). Unexpectedly, eritoran treatment further reduced the absolute counts of all the three lymphocyte populations. We next examined the relative activation status of gated CD8^+^ and CD4^+^ T cells. EBOV infection increased the percentages of CD8^+^ and CD4^+^ T cells positive for gamma interferon (IFN-γ) by 4.1-fold (Fig. 3B) and 3.7-fold (Fig. 3C), respectively. Infection did not affect the percentages of CD4^+^ T cells positive for interleukin-4 (IL-4) (Fig. 2D) or the activation marker CD69 (Fig. S5A); however, it increased percentages of IL-17A^+^ CD4^+^ T cells by 4.0-fold (Fig. 3E). Treatment with eritoran dramatically reduced the levels of IFN-γ-secreting CD8^+^ (Fig. 3B) and CD4^+^ (Fig. 3C) T cells and IL-17A-secreting CD4^+^ T cells, which nearly reached the levels in mock-infected mice (Fig. 3E). Eritoran treatment, however, increased the percentages of CD4^+^ T cells positive for IL-4 by 3.8-fold (Fig. 3D) and increased the percentages of CD4^+^ T cells (but not CD8^+^ T cells [Fig. S5B]) positive for CD69 by 2.8-fold (Fig. S5A). No effects on the percentages of B cells, NK cells, and monocytes were observed (Fig. S6). These data suggest that treatment with eritoran effectively blunts Th1 and Th17 responses and shifts the response toward Th2.

10.1128/mBio.00226-17.5 FIG S5  Percentages of CD69^+^ T lymphocytes. Percentages of CD4^+^ CD3^+^ (A) and CD8^+^ CD3 (B) T lymphocytes positive for CD69. Mean values ± standard errors based on 5 mice per group on day 6 postinfection. NS, no significant difference. Download FIG S5, PPT file, 0.2 MB. Copyright © 2017 Younan et al. 2017 Younan et al. This content is distributed under the terms of the Creative Commons Attribution 4.0 International license.

10.1128/mBio.00226-17.6 FIG S6  Analysis of peripheral blood mononuclear cell (PBMC) subsets. Percentages of CD19^+^ B cells (A), CD335^+^ NK cells (B), and CD14^+^ monocytes (C) determined by flow cytometry. Mean values ± standard errors based on 5 mice per group. NS, no significant difference. Download FIG S6, PPT file, 0.1 MB. Copyright © 2017 Younan et al. 2017 Younan et al. This content is distributed under the terms of the Creative Commons Attribution 4.0 International license.

**FIG 4:**
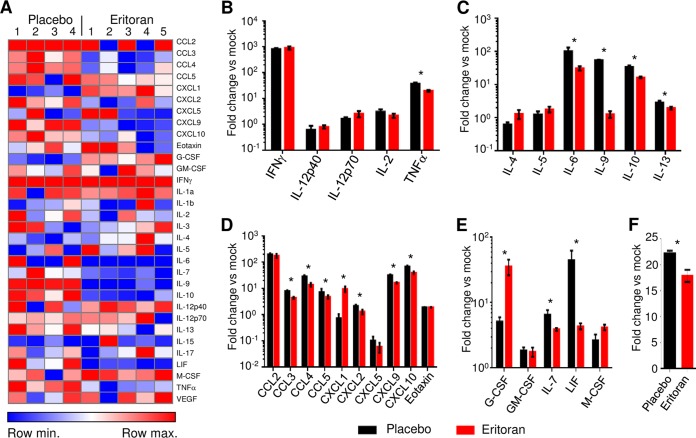
Eritoran treatment reduces cytokine storm and the levels of free radicals. (A) Heat map of serum cytokine levels in placebo- and eritoran-treated mice following normalization to mock levels, day 6 postinfection. (B to F) Serum levels of inflammatory mediators, including Th1-associated cytokines (B), Th2-associated cytokines (C), chemokines (D), cytokines associated with stem cell and progenitor differentiation and survival of T lymphocytes and NK cells (E), and reactive oxygen species/reactive nitrogen species (F). Average fold difference versus uninfected mice, mean values ± standard errors based on 5 mice per group (mock and eritoran) or 4 mice per group (placebo). *, *P* < 0.05 for eritoran compared to placebo. See also Table S1.

As indicated in Fig. 4A and Table S1, analysis of cytokine/chemokine levels using a multiplex bead-based assay resulted in distinctive patterns being observed between placebo- and eritoran-treated mice. EBOV infection resulted in an increase of most inflammatory markers assayed in both the placebo- and eritoran-treated groups in comparison to mock-infected mice. However, treatment with eritoran reduced the levels of several cytokines: whereas tumor necrosis factor alpha (TNF-α) was the only Th1-associated cytokine reduced (Fig. 4B), multiple Th2-associated cytokines, including IL-6, IL-9, IL-10, and IL-13, were reduced in eritoran-treated mice compared to mice receiving the placebo (Fig. 4C). The reduction in TNF-α may be in part due to the direct inhibitory effects of eritoran, as TNF-α is a central effector molecule associated with the activation of the TLR4 signaling pathway. The only notable cytokines that were increased in eritoran-treated mice were granulocyte colony-stimulating factor (G-CSF) and macrophage colony-stimulating factor (M-CSF). Due to the pleiotropic role of TNF-α in the inflammatory response, we assessed the susceptibility of TNFR1R2^−/−^ mice to EBOV infection. No differences in disease and survival were observed between wild-type and TNFR1R2^−/−^ mice (Fig. S1). These findings were not entirely unexpected, as although excess amounts or prolonged expression of TNF-α may contribute to pathogenesis of disease, TNF-α also plays a central role in the regulation of the immune response.

10.1128/mBio.00226-17.7 TABLE S1  Analysis of cytokines and chemokines in sera of mice infected with EBOV and treated with eritoran. Serum cytokine and chemokine levels were determined using multiplex analysis at day 6 post-EBOV infection. Results shown are averages from 4 mice for the placebo group and 5 mice for mock- and eritoran-treated groups ± standard errors. *P* values are indicated for eritoran versus placebo treatment. *, *P* < 0.05. Download TABLE S1, DOC file, 0.1 MB. Copyright © 2017 Younan et al. 2017 Younan et al. This content is distributed under the terms of the Creative Commons Attribution 4.0 International license.

Consistent with the overall downregulation of the inflammatory response associated with eritoran treatment, we detected a broad decrease in chemokine production (Fig. 4D), including a significant reduction of CCL3, CCL4, CCL5, CXCL2, CXCL9, and CXCL10. Eritoran treatment, however, did result in a significant 10.0-fold increase in the production of CXCL1, which is a neutrophil chemoattractant secreted by macrophages, epithelial cells, and activated neutrophils (19).

Eritoran treatment also affected the levels of cytokines associated with stem cell differentiation and progenitor development (Fig. 4E). Specifically, eritoran resulted in an increase in the levels of G-CSF by 7.0-fold; G-CSF stimulates differentiation of progenitor stem cells toward granulocyte development. Conversely, eritoran reduced the levels of IL-7 by 41%; IL-7 promotes hematopoietic stem cell differentiation into lymphoid progenitor cells and differentiation and survival of T cells and NK cells. The reduction in IL-7 may contribute to the overall decrease in T lymphocytes observed in eritoran-treated mice. Eritoran treatment also reduced serum levels of leukemia inhibitory factor (LIF) by 10.3-fold. This finding is particularly interesting as LIF expression levels inversely correlate with cellular differentiation (20); hence, a decrease in LIF levels in eritoran-treated mice is indicative of increased immune cell differentiation. Last, we analyzed serum samples for total levels of free radicals, including hydrogen peroxide, nitric oxide, peroxyl radical, and peroxynitrite anion. Consistent with the previous observations (21), EBOV infection increased the levels of free radicals 22.3-fold (Fig. 4F). Interestingly, eritoran treatment partially reversed this, resulting in a 23% reduction of free radicals.

**FIG 5:**
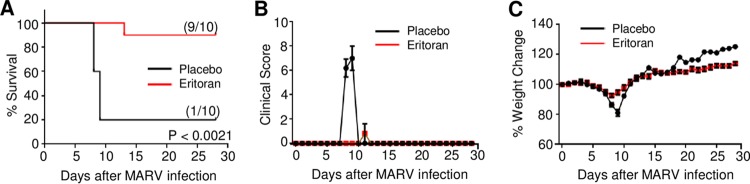
Eritoran protects mice from lethal MARV challenge. C57BL/6J mice were challenged via the i.p. route with 1,000 PFU of mouse-adapted MARV. Mice received 10 daily injections of eritoran or placebo (vehicle) via the i.p. route. (A) Survival curves generated from MARV-infected mice treated with placebo or eritoran. (B) Illness scores assigned as described in Materials and Methods. (C) Weight change following MARV challenge. Mean values from two independent experiments of 5 mice per group ± standard errors (A to C).

As filoviruses share common features associated with bacterial sepsis, we next assessed the ability of eritoran to protect mice from lethal MARV infection. Mice were infected with mouse-adapted MARV at day 0 and treated as described for Fig. 1A. As indicated in Fig. 5A, 90% of eritoran-treated mice survived lethal MARV infection, whereas in the placebo-treated group only one mouse (20% of total) survived. The average illness score for eritoran-treated mice remained relatively unchanged, as only the mouse that succumbed to infection received a score greater than 1 (Fig. 5B). Conversely, all mice in the placebo-treated group had high illness scores at days 8 and 9 postchallenge. As observed in EBOV-infected mice, the average weight of placebo-treated mice decreased considerably following MARV challenge (Fig. 5C); however, only a minimal reduction was observed in eritoran-treated mice. Similarly to the weight gains observed in EBOV-infected eritoran-treated mice, an increase in weight was observed in MARV-infected mice receiving eritoran treatment. Taken together, our data indicate that eritoran treatment is effective at promoting survival of lethal filovirus infections.

## DISCUSSION

We propose that a generalized reduction in the global release of inflammatory mediators in response to filovirus infections following eritoran treatment may alleviate pathogenic features of disease associated with an overactive immune response. In this regard, a recent study indicated that a moderate decrease of inflammatory mediators strongly correlated with survival in bacterial sepsis (22). Fatalities in EBOV infection have been associated with high levels of the proinflammatory cytokine IL-6, chemokines, and the anti-inflammatory cytokine IL-10 (6, 23); intriguingly, eritoran reduced both IL-6 and IL-10 in these studies, in addition to inhibiting chemokine production (Fig. 3C and D).

The observed reduction in the inflammatory response is likely directly associated with the known inhibitory activity of eritoran, which is a well-characterized, TLR4-specific inhibitor. TLR4 is expressed in numerous cell types, including both immune (e.g., both adaptive and innate immune cell subsets) and nonimmune (e.g., intestinal epithelial cell lines) cells (24). Hence, the broad reduction of inflammatory mediators may be due to the global effects of eritoran. As demonstrated in bacterial sepsis, continued TLR4 stimulation results in an overexacerbated immunological response, which ultimately elicits a more damaging than beneficial response (25). As we observed in these studies using TLR4^−/−^ mice, the absence of TLR4 following EBOV infection is equally detrimental (see Fig. S1 in the supplemental material). This finding suggests that the effects of eritoran may partially blunt TLR4 signal transduction induced by EBOV; hence, a more limited induction of the TLR4 signaling pathway may result in beneficial immune responses.

During sepsis, the burst of inflammatory mediators (e.g., TNF-α) increases membrane rigidity of neutrophils, which limits tissue migration of neutrophils. Targeting of TNF-α in animal models of bacterial sepsis led to a decrease of inflammatory mediators (26). In this study, the lower number of neutrophils in peripheral blood of eritoran-treated mice may be due to increased tissue migration, increased turnover, or reduced chemotactic signaling resulting in a decrease of neutrophil mobilization from the bone marrow. Similarly, lower levels of T lymphocytes in peripheral blood of eritoran-treated mice may be the result of increased tissue migration due to the development of improved chemokine gradients.

It is possible that in addition to blocking GP-mediated stimulation of TLR4, eritoran also protects EBOV-infected mice by reducing the inflammatory effects of Ox-PLs. Ox-PLs, which are generated following infections, apoptosis, and tissue damage, have been shown to interfere with maturation of dendritic cells, resulting in limited lymphocyte activation (27), and to trigger production of both pro- and anti-inflammatory mediators (28, 29). It is feasible that Ox-PLs may further stimulate TLR4 signaling during the course of EVD. This hypothesis is supported by previous observations utilizing an influenza model of disease, which demonstrated a reduction in IL-6 following eritoran antagonism of Ox-PLs by blocking interaction with TLR4 (13).

Interestingly, unlike lethal influenza virus, which failed to cause lethal infection in TLR4^−/−^ mice (13), EBOV appeared to be equally lethal in wild-type (wt) and TLR4^−/−^ mice (Fig. S1). This difference is likely to be related to the very different pathogenesis of the disease caused by the two viruses. Although both viruses are known to induce a severe cytokine storm, influenza virus causes localized infections of respiratory epithelium, while filoviruses cause systemic infections with virus infecting almost all types of cells and disseminating to almost all tissues. The survival of TLR4^−/−^ mice infected with lethal influenza virus suggests that the cytokine storm associated with TLR4 signaling is a central factor contributing to mortality. In contrast, the lack of survival of TLR4^−/−^ mice infected with EBOV suggests that while the TLR4-mediated cytokine storm contributes to mortality, TLR4 signaling also contributes to the protective immune response.

The delayed treatment with eritoran starting at 48 h postinfection failed to result in any survival (Fig. S2D to F). We note that while infection of mice with EBOV typically results in death at around day 6, the onset of symptoms in EBOV-infected humans can range from day 2 to 21 after exposure (30), with death occurring an average of 6 to 16 days after the onset of symptoms (31). We also note that we used the 1,000-PFU EBOV challenge dose, while in nonhuman primate (NHP) models of filovirus infections infectious doses of less than 10 PFU are lethal (32, 33). Clearly, regimens of eritoran treatments of EBOV infection need to be tested in NHPs. Even if only early treatment of NHPs is successful, it will still be highly useful for accidental exposures of health care personnel and laboratory workers. While treatment of EBOV infection with monoclonal antibodies has been demonstrated to be effective (34), it is highly specific to a filoviral species. Unlike antibody treatment, eritoran treatment is not specific to a filoviral species, affects the disease pathogenesis rather than the virus, and presumably can be used in combination with antibodies.

A recent study demonstrated that survival of bacterial sepsis correlates with a moderate decrease of inflammatory mediators (22), which parallels these findings. The global reduction in inflammatory mediators indicates that eritoran blunts the development of a cytokine storm. Furthermore, bacterial infections causing sepsis are typically characterized by a shift from a Th1 response to a Th2 response as disease progresses (35), which may also be the case for filovirus infection. The extracellular milieu in eritoran-treated mice is suggestive of an extended Th1 response in comparison to serum cytokine analysis of the placebo group, which appears to have progressed to a Th2 response. Conversely, intracellular staining analysis indicates that an opposing, Th2 response was being initiated at day 6 in eritoran-treated mice as determined by an increase in IL-4-secreting CD4^+^ T cells in comparison to untreated, EBOV-infected mice. This can be explained by eritoran treatment delaying initiation of the Th2 response, as noted by the significant decrease in Th2-associated cytokines (Fig. 4C). Patients with severe bacterial sepsis have a low Th1/Th2 ratio, whereas the opposite is observed in nonseptic patients (36); hence, the reduced plasma viremia and improved survival with eritoran treatment may be due to a prolonged Th1 response. Further, in-depth studies in nonhuman primate models are needed to validate the effects of eritoran treatment on the kinetics of Th-associated responses. These studies would enable extended sample collection and therefore provide more detailed analysis of cellular and inflammatory mediators. Overall, these studies provide the most compelling evidence to date regarding the similarities between filovirus infection and those observed in classical bacterial sepsis. The well-documented safety profile and the findings here warrant further investigation of eritoran as a potential therapy against EBOV in a nonhuman primate model of EBOV and in clinical trials.

## MATERIALS AND METHODS

### Work in BSL-4 containment facilities.

All work with EBOV and MARV was performed in biosafety level 4 (BSL-4) facilities of the Galveston National Laboratory. Flow cytometry was performed following inactivation with 4% paraformaldehyde in phosphate-buffered saline (PBS) for 48 h according to the University of Texas Medical Branch (UTMB) standard operating procedure and removed from BSL-4 conditions for analysis with an LSRII Fortessa flow cytometer (BD Biosciences) available at the UTMB Flow Cytometry Core Facility. To remove serum samples from EBOV-infected mice, samples were gamma irradiated with a 5-Mrad dose according to the UTMB standard operating procedure protocol.

### EBOV and MARV infections of mice and treatment with eritoran.

EBOV and MARV infections of mice were performed in the animal BSL-4 (ABSL-4) containment facilities of the Galveston National Laboratory. The animal protocols for testing of eritoran in mice were approved by the Institutional Animal Care and Use Committee of the UTMB. Eight- to 10-week-old wild-type C57BL/6 mice, TLR4^−/−^ mice, strain B6.B10ScN-TLR4^lps-del^/JthJ (catalog no. 007227; The Jackson Laboratory) and TNFR1/2^−/−^ mice, strain B6.129S-Tnfrsf1a^tm1/mx^Tnfrs1b^tm1/mx^/J (catalog no. 003243; The Jackson Laboratory), were infected with 1,000 PFU of either mouse-adapted EBOV strain Mayinga (17) or mouse-adapted MARV strain Ci67 (37) by intraperitoneal injection. All virus stocks were back titrated at time of infection to verify viral titers. Eritoran was prepared as described by the manufacturer and diluted to a final concentration of 2.33 mg/ml (Eisai). Mice received daily administration consisting of 100 μl of placebo (vehicle) or eritoran at an effective dose of 233 μg/day. Mice were monitored twice daily from day 0 to day 14 postchallenge, followed by once-daily monitoring from day 15 to the end of the study at day 28. The disease was scored using the following parameters: dyspnea (possible scores of 0 to 5), recumbency (0 to 5), unresponsiveness (0 to 5), and bleeding/hemorrhage (0 to 5). All mice were euthanized at day 28 post-EBOV challenge.

### Analysis of viremia.

Total RNA was isolated from serum samples taken at day 6 post-EBOV challenge using the QIAamp viral RNA minikit per the manufacturer’s protocol (Qiagen). EBOV was quantified with the One-Step reverse-transcription (RT) droplet digital PCR (ddPCR) advanced kit for probes (Bio-Rad), with probes specific for the NP gene fragment corresponding to nucleotides 2095 to 2153 of EBOV genomic RNA (GenBank accession number AF086833) using forward primer GCCACTCACGGACAATGACA, reverse primer GCATGCGAGGGCTGGTT, and probe 6-carboxyfluorescein (FAM)–AGAAATGAACCCTCCGGCT-MGB. Briefly, 50 pg of RNA was added to 5 µl of SuperMix, 2 µl of reverse transcriptase enzyme, 1 µl of 300 mM dithiothreitol (DTT), and 1 µl of 20× NP custom TaqMan assay (Life Technologies, Inc.) for each sample. ddPCR mixtures were loaded onto cartridges to create droplets on a QX200 droplet generator (Bio-Rad). The droplets were transferred onto 96-well PCR plates (Eppendorf) and amplified on a C1000 thermal cycler with a 96-deep-well reaction module (Bio-Rad). The following reaction conditions were used: 42°C for 60 min and 95°C for 10 min, followed by 39 cycles of 95°C for 15 s and 60°C for 1 min, and a final enzyme deactivation step of 98°C for 10 min. Finally, the PCR plates were loaded onto a droplet reader, which quantifies the number of positive and negative droplets in each sample. Analysis was performed using QuantaSoft software to get the final concentrations in each sample.

### Analysis of neutrophils and T cells in peripheral blood by flow cytometry.

Erythrocytes were lysed using lytic lysis buffer (Sigma-Aldrich) as recommended by the manufacturer. Cells were pelleted at 400 × *g*, washed in phosphate-buffered saline containing 2% fetal bovine serum, and stained with the following antibodies: panel 1, CD3-BUV.395 (145-2C11; BD Biosciences), CD4-peridinin chlorophyll protein (PerCP).Cy5.5 (RM4-5; BD Biosciences), CD8-BVLT.421 (53-6.7; BD Biosciences), IFN-γ–Alexa.488 (XMG1.2; BD Biosciences), IL-17A–phycoerythrin (PE) (TC11-18H10.1; BD Biosciences), and IL-4–allophycocyanin (APC) (11B11; BD Biosciences); panel 2, CD11b-Alexa.488 (M1/70; BioLegend), Ly6G/Ly6C-Alexa.647 (RB6-8C5; BioLegend), CD64-BVLT.421 (X54-5/7.1; BioLegend), CD69-PE (H1.2F3; BioLegend), CD3-BUV.395, and CD4-PerCP.Cy5.5; panel 3, CD19-APC (1D3; BD Biosciences), CD14-BUV.737 (rmC5-3; BD Biosciences), and CD335-fluorescein isothiocyanate (FITC) (29A1.4; BD Biosciences). For determination of absolute counts, CountBright absolute counting beads (Thermo Fisher Scientific) were used per the manufacturer’s instructions. Data were collected using an LSRII Fortessa flow cytometer (BD Biosciences) and analyzed with FlowJo.

### Analysis of cytokines, chemokines, and free radicals in the peripheral blood.

Serum was collected on day 6 post-EBOV infection. Samples were gamma irradiated with the 5-Mrad dose according to the UTMB standard operating procedure protocol, removed from the BSL-4 facility, and analyzed using a Multiplex-32 magnetic bead-based assay (Millipore) by Eve Technologies. Total free radicals were measured using the OxiSelect *in vitro* ROS/reactive nitrogen species (RNS) assay kit (Cell Biolabs) using the protocol provided by the manufacturer.

### Statistical analysis.

Analysis was performed using GraphPad Prism (version 6.04) (GraphPad Software, Inc.). Comparison of survival curves was conducted using a log rank (Mantel-Cox) test. A paired one-sided *t* test was used to compare the levels of viremia, cytokines, chemokines, and free radicals in plasma and differences in the percentages of immune cells between groups.

### Data availability.

Any additional information regarding materials and methods will be provided upon request.
