# Supplementary material for: The Toll-Like Receptor 4 Antagonist Eritoran Protects Mice from Lethal Filovirus Challenge
Source: mBio. 2017 Apr 25;8(2):e00226-17. doi: 10.1128/mBio.00226-17 (PMC5405229; doi:10.1128/mBio.00226-17)
Supplement: FIG S1 [file mbo002173286sf1.ppt]

## Slide 1
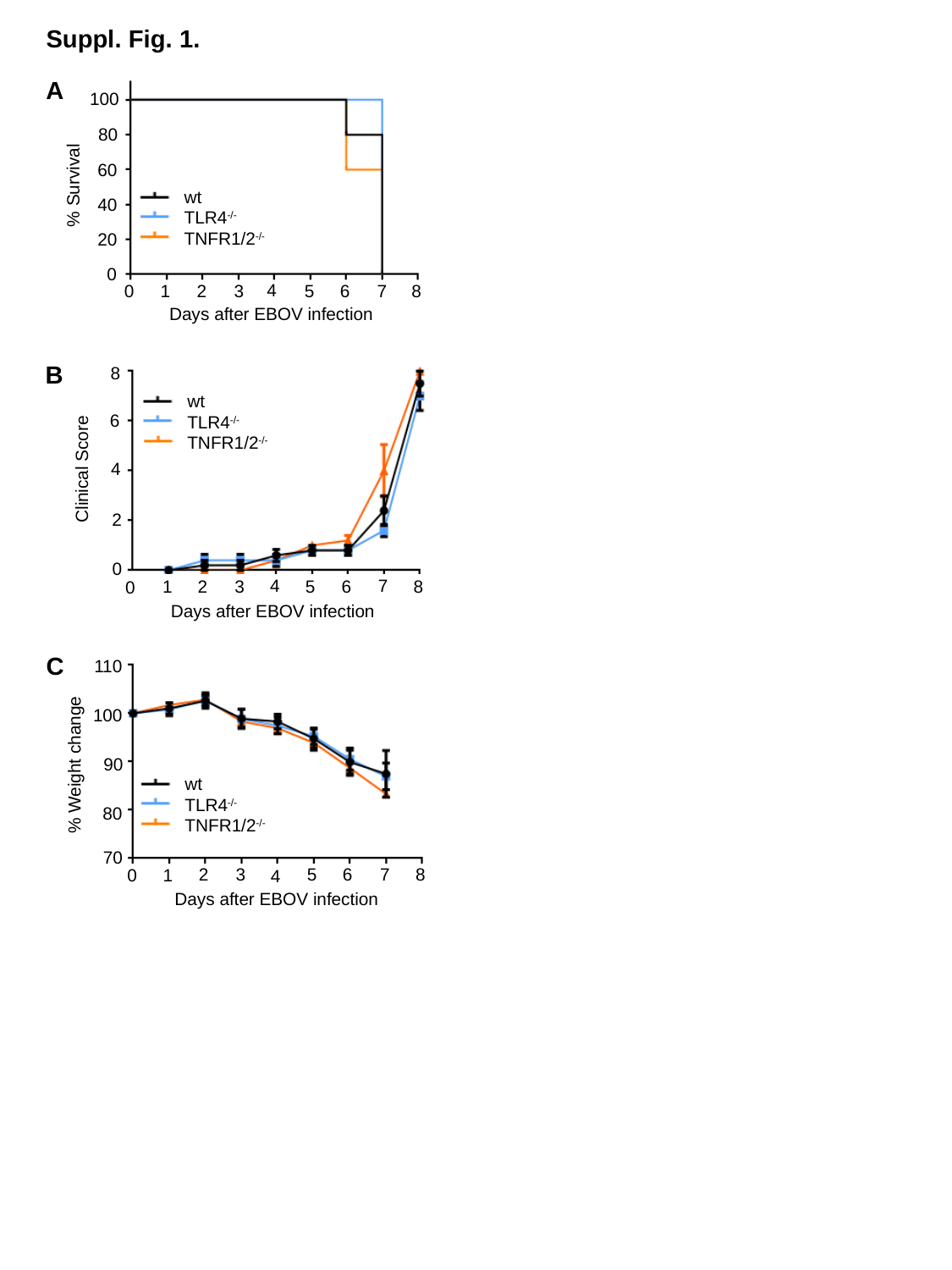

Suppl. Fig. 1.
A
100
80
60
% Survival
wt
TLR4-/-
TNFR1/2-/-
40
20
0
0
4
5
6
7
8
0
1
2
3
Days after EBOV infection
B
 8
wt
TLR4-/-
TNFR1/2-/-
 6
 4
Clinical Score
 2
 0
 4
 7
 2
 3
 5
 6
 8
 1
 0
Days after EBOV infection
C
110
100
90
% Weight change
wt
TLR4-/-
TNFR1/2-/-
80
70
 7
 8
 2
 3
 5
 6
 1
 0
 4
Days after EBOV infection
