# Supplementary material for: The Toll-Like Receptor 4 Antagonist Eritoran Protects Mice from Lethal Filovirus Challenge
Source: mBio. 2017 Apr 25;8(2):e00226-17. doi: 10.1128/mBio.00226-17 (PMC5405229; doi:10.1128/mBio.00226-17)
Supplement: FIG S2 [file mbo002173286sf2.ppt]

## Slide 1
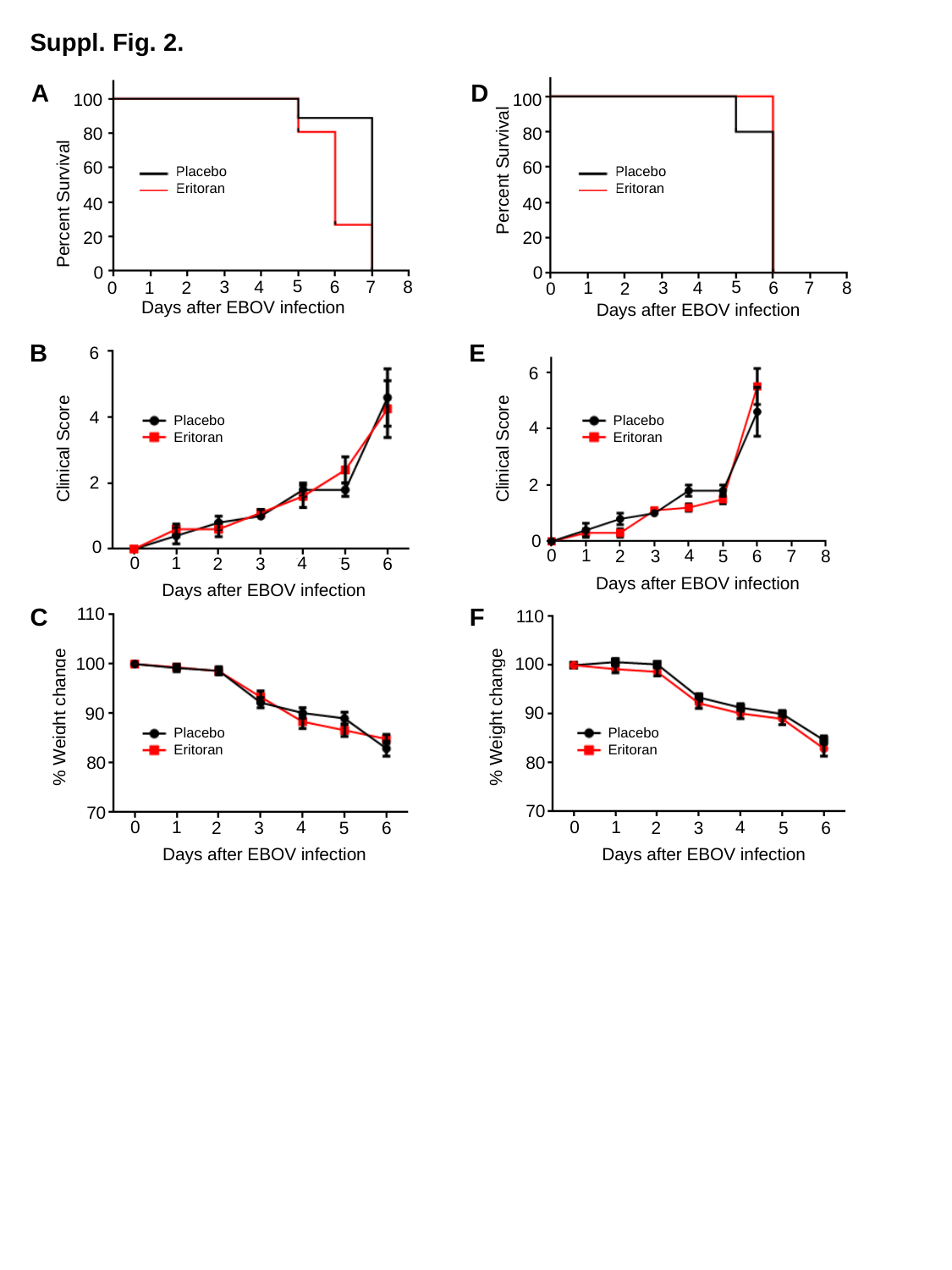

Suppl. Fig. 2.
A
D
100
100
80
80
60
60
Percent Survival
Placebo
Eritoran
Placebo
Eritoran
40
40
Percent Survival
20
20
0
0
 5
 3
 4
 6
 7
 5
 8
 1
 3
 4
 6
 7
 0
 2
 8
 1
 0
 2
Days after EBOV infection
Days after EBOV infection
B
E
6
6
4
Placebo
Eritoran
Placebo
Eritoran
4
Clinical Score
Clinical Score
2
2
0
0
 0
 1
 4
 2
 3
 5
 6
 7
 8
 0
 1
 4
 2
 3
 5
 6
Days after EBOV infection
Days after EBOV infection
C
F
110
110
100
100
90
90
% Weight change
% Weight change
Placebo
Eritoran
Placebo
Eritoran
Placebo
Eritoran
80
80
70
70
 0
 1
 4
 0
 1
 4
 2
 3
 5
 6
 2
 3
 5
 6
Days after EBOV infection
Days after EBOV infection
