# Supplementary material for: The Toll-Like Receptor 4 Antagonist Eritoran Protects Mice from Lethal Filovirus Challenge
Source: mBio. 2017 Apr 25;8(2):e00226-17. doi: 10.1128/mBio.00226-17 (PMC5405229; doi:10.1128/mBio.00226-17)
Supplement: FIG S3 [file mbo002173286sf3.ppt]

## Slide 1
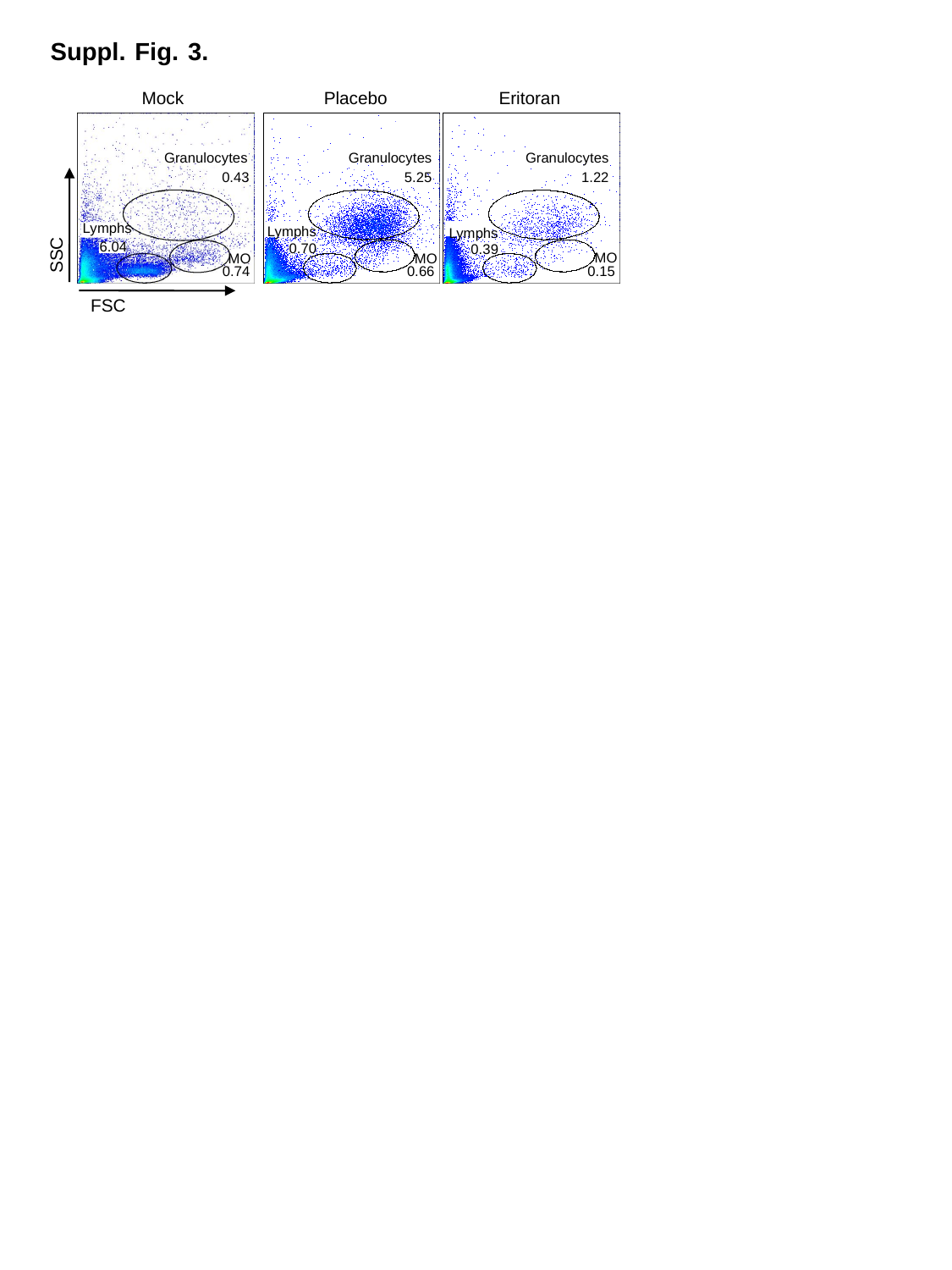

Suppl. Fig. 3.
Mock
Placebo
Eritoran
Granulocytes
0.43
Lymphs
6.04
MO
0.74
Granulocytes
5.25
Lymphs
0.70
MO
Granulocytes
1.22
Lymphs
0.39
MO
0.15
SSC
0.66
FSC
