# Supplementary figures and images for: The Toll-Like Receptor 4 Antagonist Eritoran Protects Mice from Lethal Filovirus Challenge
Source: mBio. 2017 Apr 25;8(2):e00226-17. doi: 10.1128/mBio.00226-17 (PMC5405229; doi:10.1128/mBio.00226-17)

## Slide 1
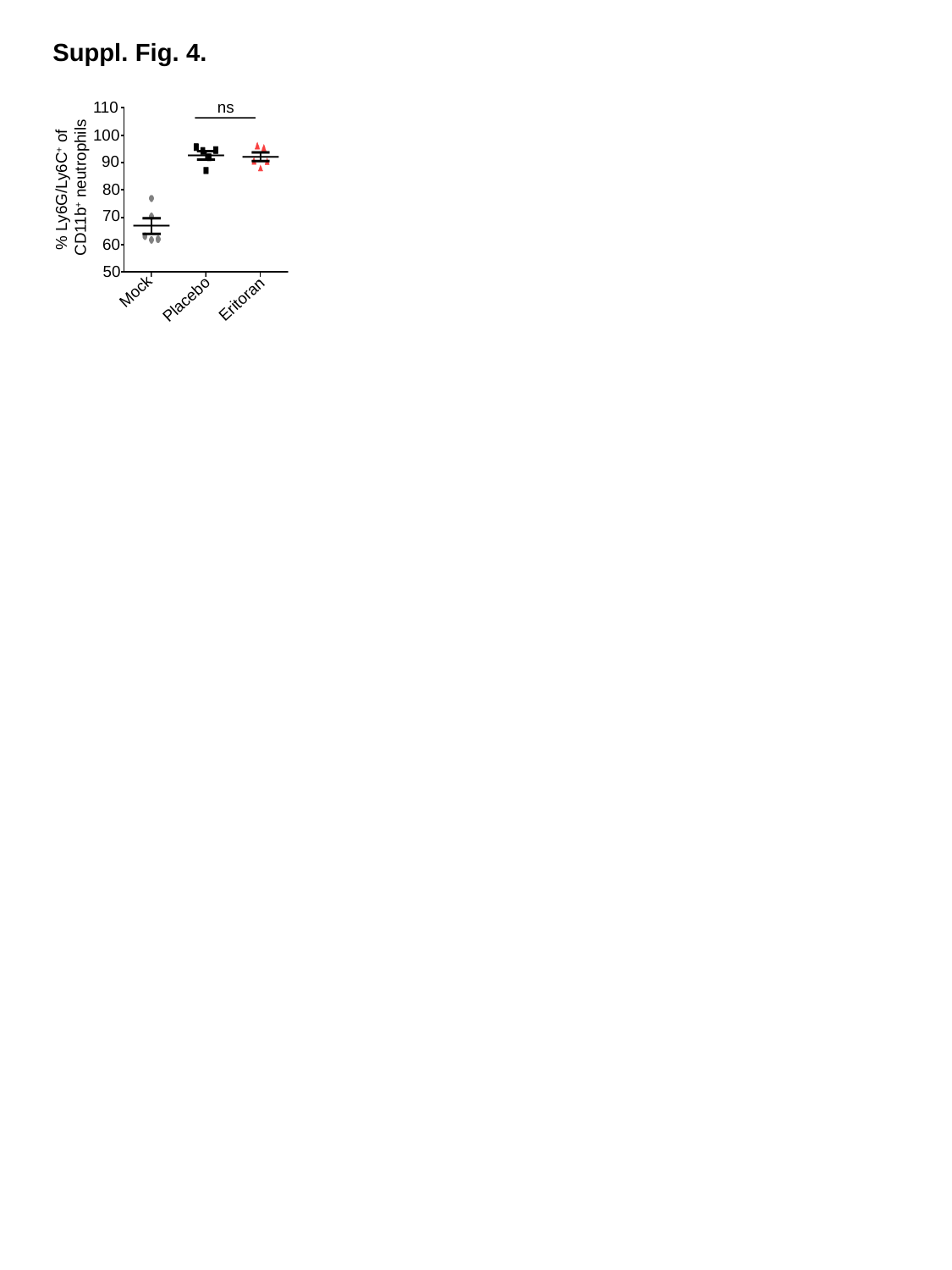

Suppl. Fig. 4.
110
ns
100
90
% Ly6G/Ly6C+ of
CD11b+ neutrophils
80
70
60
50
Mock
Placebo
Eritoran

Supplement: FIG S4 [file mbo002173286sf4.ppt]
