# Supplementary material for: The Toll-Like Receptor 4 Antagonist Eritoran Protects Mice from Lethal Filovirus Challenge
Source: mBio. 2017 Apr 25;8(2):e00226-17. doi: 10.1128/mBio.00226-17 (PMC5405229; doi:10.1128/mBio.00226-17)
Supplement: FIG S5 [file mbo002173286sf5.ppt]

## Slide 1
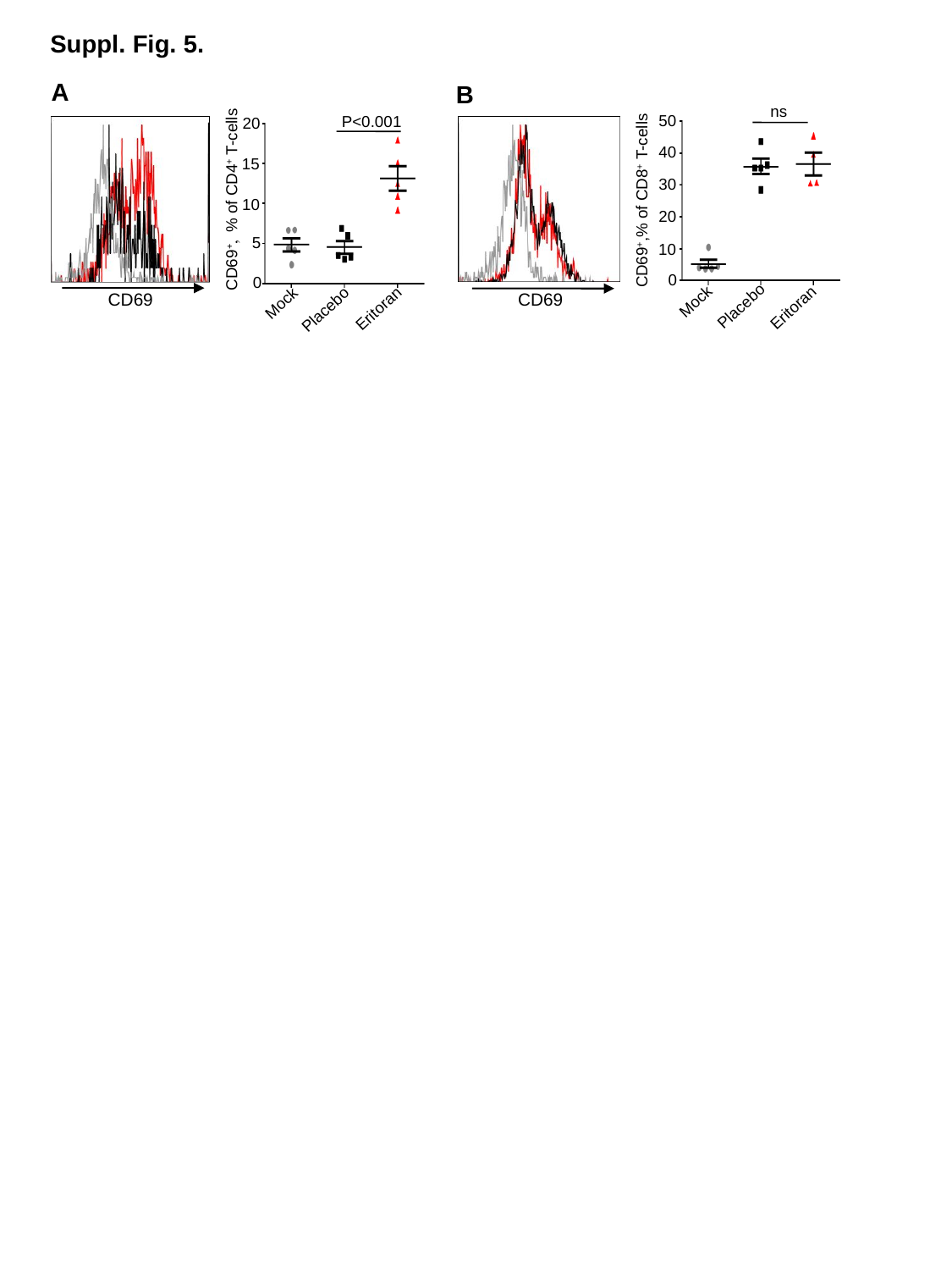

Suppl. Fig. 5.
A
B
ns
50
P<0.001
20
40
15
30
CD69 % of CD4+ T-cells
CD69+,% of CD8+ T-cells
10
20
5
10
0
0
CD69
CD69
Mock
Mock
Eritoran
Placebo
Placebo
Eritoran
