# Supplementary material for: The Toll-Like Receptor 4 Antagonist Eritoran Protects Mice from Lethal Filovirus Challenge
Source: mBio. 2017 Apr 25;8(2):e00226-17. doi: 10.1128/mBio.00226-17 (PMC5405229; doi:10.1128/mBio.00226-17)
Supplement: FIG S6 [file mbo002173286sf6.ppt]

## Slide 1
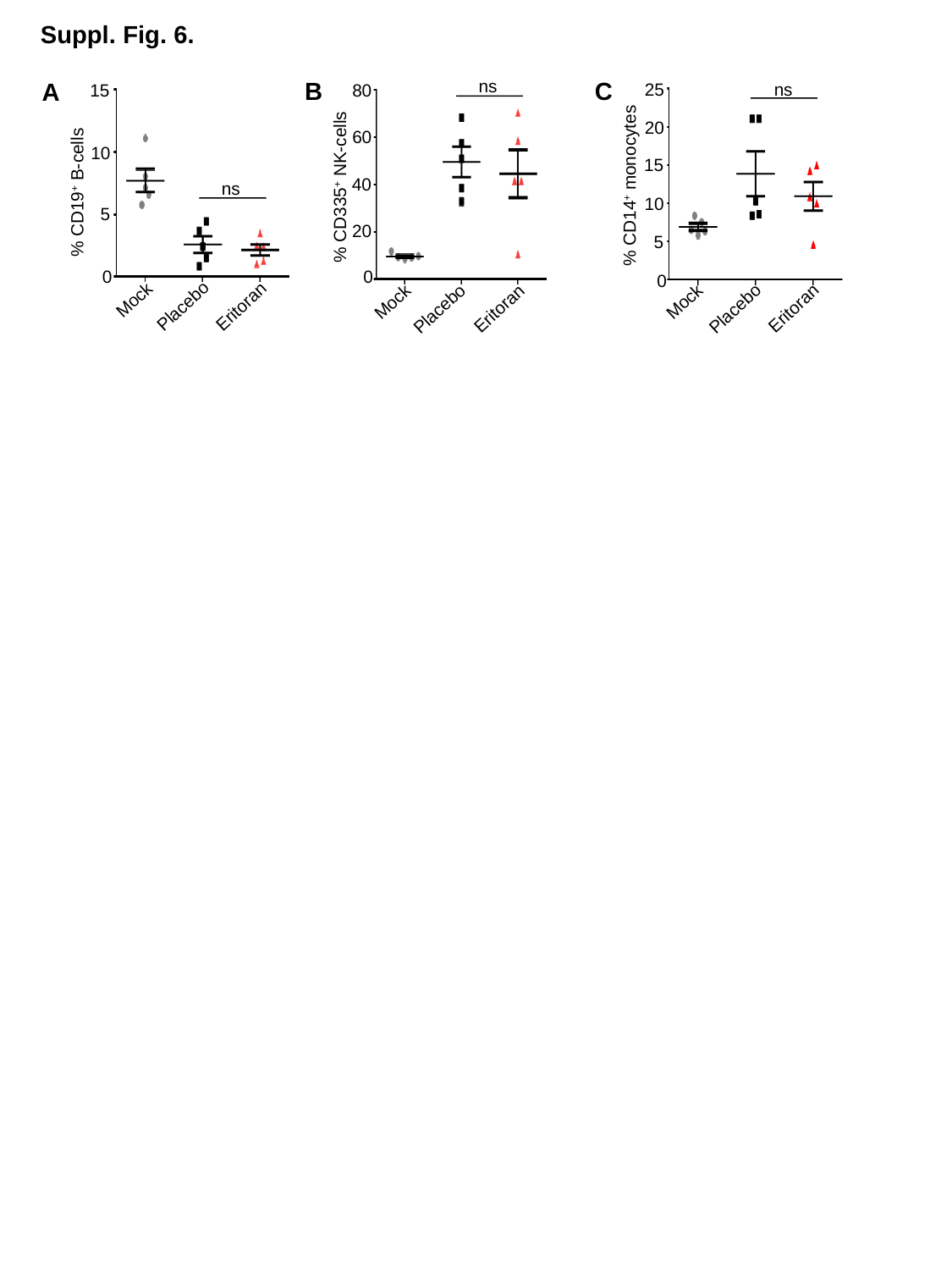

Suppl. Fig. 6.
ns
B
C
A
25
ns
15
80
20
60
10
15
40
% CD14+ monocytes
% CD335+ NK-cells
ns
% CD19+ B-cells
10
5
20
 5
0
0
0
Mock
Mock
Mock
Placebo
Eritoran
Placebo
Eritoran
Placebo
Eritoran
