# Supplementary material for: The Toll-Like Receptor 4 Antagonist Eritoran Protects Mice from Lethal Filovirus Challenge
Source: mBio. 2017 Apr 25;8(2):e00226-17. doi: 10.1128/mBio.00226-17 (PMC5405229; doi:10.1128/mBio.00226-17)
Supplement: TABLE S1 [file mbo002173286st1.doc]

**Supplemental Table 1.** Analysis of cytokines and chemokines in sera of mice infected with EBOV and treated with eritoran.

| **Cytokine/**  **Chemokine** | **Mock** | **Placebo** | **Eritoran** | **P-value** |  |
| --- | --- | --- | --- | --- | --- |
| CCL2 | 36.6 ± 8.7 | 7504.8 ± 728.6 | 6633.1 ± 1131.9 | 0.562 | |
| CCL3 | 75.7 ± 11.5 | 602.4 ± 57.9 | 339.9 ± 29.8 | 0.003* | |
| CCL4 | 62.7 ± 0.1 | 1849.7 ± 160.1 | 888.0 ± 134.3 | 0.002* | |
| CCL5 | 39.9 ± 2.2 | 394.4 ± 37.2 | 194.7 ± 27.9 | 0.005* | |
| CXCL1 | 82.4 ± 10.1 | 81.1 ± 24.1 | 806.2 ± 194.1 | 0.031* | |
| CXCL2 | 161.0 ± 48.5 | 350.8 ± 42.6 | 211.8 ± 40.4 | 0.050* | |
| CXCL5 | 4782.0 ± 980.2 | 663.1 ± 180.7 | 483.7 ± 40.0 | 0.386 | |
| CXCL9 | 82.0 ± 15.6 | 2629.6 ± 204.2 | 1363.9 ± 71.2 | 0.0003* | |
| CXCL10 | 98.6 ± 7.7 | 6827.8 ± 697.2 | 4033.9 ± 404.1 | 0.008* | |
| Eotaxin | 493.5 ± 47.8 | 942.4 ± 16.6 | 944.0 ± 30.7 | 0.966 | |
| G-CSF | 285.6 ± 23.5 | 1464.9 ± 239.9 | 10245.0 ± 2762.3 | 0.027* | |
| GM-CSF | 28.4 ± 4.8 | 52.5 ± 6.2 | 50.3 ± 8.2 | 0.845 | |
| IFNγ | <1.0 ± 0.0 | 824.2 ± 81.7 | 919.9 ± 134.0 | 0.587 | |
| IL1α | 125.1 ± 42.4 | 151.7 ± 31.6 | 166.8 ± 10.8 | 0.632 | |
| IL1β | 23.1 ± 3.8 | 40.1 ± 5.5 | 45.3 ± 16.6 | 0.806 | |
| IL2 | 14.3 ± 1.9 | 44.8 ± 10.7 | 32.0 ± 6.1 | 0.339 | |
| IL3 | 1.2 ± 0.2 | 2.9 ± 1.2 | 2.2 ± 0.7 | 0.613 | |
| IL4 | 0.8 ± 0.4 | 0.5 ± 0.1 | 1.0 ± 0.3 | 0.18 | |
| IL5 | 21.2 ± 6.0 | 26.7 ± 6.4 | 38.6 ± 7.5 | 0.283 | |
| IL6 | 4.1 ± 0.6 | 425.9 ± 126.6 | 130.3 ± 18.3 | 0.034* | |
| IL7 | 11.5 ± 3.9 | 75.6 ± 13.0 | 44.9 ± 2.1 | 0.034* | |
| IL9 | <1.0 ± 0.0 | 56.1 ± 1.7 | 1.3 ± 0.3 | <0.0001* | |
| IL10 | 9.1 ± 1.7 | 317.5 ± 30.4 | 152.3 ± 9.5 | 0.0007* | |
| IL12p40 | 48.7 ± 6.8 | 30.2 ± 13.3 | 54.5 ± 18.5 | 0.479 | |
| IL12p70 | 25.4 ± 13.1 | 43.2 ± 6.6 | 54.5 ± 18.5 | 0.621 | |
| IL13 | 24.6 ± 4.2 | 72.3 ± 8.2 | 48.5 ± 4.5 | 0.030* | |
| IL15 | 70.6 ± 17.4 | 148.9 ± 21.6 | 147.8 ± 14.5 | 0.97 | |
| IL17 | 0.6 ± 0.1 | 2.1 ± 0.5 | 2.2 ± 0.8 | 0.881 | |
| LIF | 0.9 ± 0.5 | 41.9 ± 16.7 | 4.1 ± 0.4 | 0.037* | |
| M-CSF | 9.2 ± 5.3 | 24.4 ± 5.6 | 38.5 ± 3.8 | 0.067 | |
| TNFα | 2.5 ± 1.6 | 94.5 ± 8.4 | 50.2 ± 3.7 | 0.001* | |
| VEGF | 0.8 ± 0.1 | 1.2 ± 0.1 | 1.3 ± 1.3 | 0.633 | |

Serum cytokine and chemokine levels were determined using multiplex analysis at day 6 post EBOV infection. Results shown are average of 4 mice for the placebo group and 5 mice for mock and eritoran treated groups ± SE. P-values are indicated for eritoran versus placebo treatment. *, P<0.05.
